# A novel behavioural approach to detecting tinnitus in the guinea pig

**DOI:** 10.1016/j.jneumeth.2012.12.023

**Published:** 2013-03-15

**Authors:** Joel I. Berger, Ben Coomber, Trevor M. Shackleton, Alan R. Palmer, Mark N. Wallace

**Affiliations:** MRC Institute of Hearing Research, University Park, Nottingham, UK

**Keywords:** Tinnitus, Guinea pig, Preyer reflex, Pinna reflex, Gap detection, Prepulse inhibition, Sodium salicylate, Whole-body startle

## Abstract

Tinnitus, the perception of sound in the absence of an external stimulus, is a particularly challenging condition to demonstrate in animals. In any animal model, objective confirmation of tinnitus is essential before we can study the neural changes that produce it. A gap detection method, based on prepulse inhibition of the whole-body startle reflex, is often used as a behavioural test for tinnitus in rodents. However, in the guinea pig the whole-body startle reflex is subject to rapid habituation and hence is not an ideal behavioural measure. By contrast, in this species the Preyer or pinna reflex is a very reliable indicator of the startle response and is much less subject to habituation. We have developed a novel adaptation of the gap detection paradigm, which uses the Preyer reflex to measure the startle response, rather than whole-body movement. Using this method, we have demonstrated changes in gap detection, in guinea pigs where tinnitus had been induced by the administration of a high dose of salicylate. Our data indicate that the Preyer reflex gap detection method is a reliable test for tinnitus in guinea pigs.

## Highlights

► Prepulse inhibition can be reliably and robustly measured using the Preyer reflex. ► The Preyer reflex is a more reliable response than the whole-body startle in guinea pigs. ► Salicylate impairs gap detection at specific background noise frequencies, indicating the presence of tinnitus. ► Measuring gap detection using the Preyer reflex is a suitable method for identifying tinnitus.

## 1 Introduction

Tinnitus is estimated to chronically affect 10–15% of the UK population at some point in their life, and in severe cases is linked to depression and even suicide (Heller, 2003). No effective cure for tinnitus has been developed to date, in part because the pathophysiological mechanisms responsible are still not understood. The development of animal models of tinnitus has played an essential role in furthering our knowledge of the physiological and molecular mechanisms that underlie this condition (Kaltenbach, 2011). The most common trigger for the condition is repeated exposure to excessive loud noise (Eggermont and Roberts, 2004), but among others it can also occur as a result of the effects of ototoxic drugs such as sodium salicylate or quinine (Ralli et al., 2010). Sodium salicylate is frequently the cause of tinnitus when patients receive high doses of aspirin (Cazals, 2000). Salicylate has been shown to reliably induce transient tinnitus in an animal model, often with less variability than noise exposure (Stolzberg et al., 2012) and over a much shorter time-scale (5 h as opposed to 8 weeks). The neural mechanisms are likely to be different to those observed following noise exposure, but it is still a useful experimental model for tinnitus.

In 1988, Jastreboff et al. developed the first behavioural test for tinnitus in rats that relied on a conditioned response; this test has since been refined (Bauer and Brozoski, 2001; Heffner and Harrington, 2002). In 2006, Turner et al. devised a new test for tinnitus that did not rely on a conditioned response, but used the whole-body startle (WBS) reflex which did not require time-consuming training periods. Moreover, this test did not rely on memory formation, a potential confound in studies of the origins and treatment of tinnitus, and enabled tinnitus-related changes to be tracked over time as behavioural extinction is not a factor. As previous work demonstrated a lag between acoustic trauma and tinnitus manifestation, in some cases as much as 8–9 weeks (Turner et al., 2006), this is a useful characteristic of the model.

**Fig. 1:**
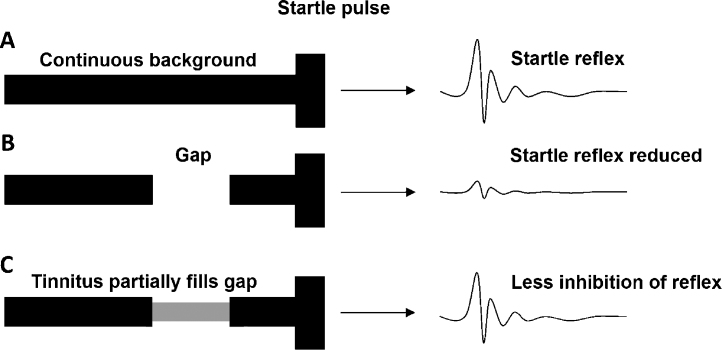
Schematic of the gap detection test, adapted from Turner and Parrish (2008). A startling pulse in continuous background noise elicits a startle reaction (A). When a gap (50 ms) is presented before the startling pulse, this reduces the amplitude of the startle response (B). When an animal is experiencing tinnitus, it will have difficulty detecting the gap, as this will be partially filled in by the tinnitus, and will show less inhibition of the startle response (C).

The paradigm devised by Turner et al. (2006) was based on the phenomenon of prepulse inhibition (PPI). A loud sound causes an animal to jump: the whole body startle (WBS) reflex. When the startling sound is cued by a preceding sound, the magnitude of the startle response is reduced (Hoffman and Searle, 1965). In the tinnitus test (Turner et al., 2006), the startle pulse was always preceded by a quiet narrow-band continuous noise (Fig. 1A). This does not alter the magnitude of the WBS, but introducing a short silent gap provides a cue for the startle pulse and PPI occurs (Fig. 1B). They assessed PPI in rats with acoustic trauma and, by varying the centre frequency of the narrow-band background noise, demonstrated selective deficits in PPI that they reasoned were due to tinnitus – with a similar spectrum to the background noise – filling the gap preceding the startle stimulus and obscuring the preceding cue (Fig. 1C); this method was validated through comparison with an operant conditioning model (as used by Bauer and Brozoski, 2001).

Although the rat has been widely used as an animal model of tinnitus, introducing further animal models will provide a broader understanding of the mechanisms leading to tinnitus, across a range of mammals, including humans. The auditory system of guinea pigs is quite typical of most mammals and their low frequency hearing sensitivity is very similar to that of humans (Prosen et al., 1978; West, 1985) providing a good species for comparison (Harrison et al., 1981).

Guinea pigs are difficult to train in complex behavioural tasks, rendering the use of the tinnitus test developed by Jastreboff et al. (1988), or that of Bauer and Brozoski (2001) or Heffner and Harrington (2002), highly problematic in this species. Although a number of studies have demonstrated WBS PPI deficits in rats, only two studies have thus far used the method for measuring deficits in guinea pigs (Dehmel et al., 2012a,b). Like the WBS, the auditory-evoked pinna or Preyer reflex is an unconscious response (Bohmer, 1988). The neural circuitry mediating the Preyer reflex has also been defined; the cochlear nucleus (CN), superior olivary complex, and the inferior colliculus (IC) all play a role, as well as regions of the reticular nucleus and facial motor nuclei (Li and Frost, 1996). Furthermore, the variables that can change the reflex are well-understood, *e.g.*, the length of the gap, delay between gap and startle stimulus, or drug intervention (Davis et al., 1993; Leitner et al., 1993; Swerdlow et al., 2001). We hypothesised that utilising the Preyer reflex to quantify startle response and gap detection may provide a useful alternative to the WBS in this behavioural test for tinnitus. Often used as a gross measure of hearing in rodents (Jero et al., 2001), the parameters involved in modulating this response are well-established. Furthermore, the Preyer reflex is susceptible to change induced by PPI (Cassella and Davis, 1986). We demonstrate that in guinea pigs the Preyer reflex is a more reliable and consistent measure of PPI than WBS.

## 2 Materials and methods

### 2.1 Animals

All procedures were carried out in accordance with the Animals (Scientific Procedures) Act 1986, UK and the approval of the ethical review committee at the University of Nottingham, UK. Experiments were conducted on a total of 24 male and female pigmented guinea pigs (GPs) from an in-house colony weighing 300–500 g at onset of behavioural testing. GPs were group-housed on a 12:12 h light:dark cycle, and food and water were freely available.

### 2.2 Measuring whole-body startle

The ability of a GP to detect a gap in background noise – and consequently produce PPI – was assessed by quantifying whole-body movement in response to the startling stimulus. GPs were placed in a wire cage (310 mm × 155 mm × 155 mm) on a custom-made startle platform in a sound proof booth; GPs were not restrained and were free to move around the cage. The startle platform was connected to a load-cell (3 kg capacity; Model 1022, Vishay Tedea-Huntleigh, Basingstoke, UK) to measure the downward force applied to the platform following a startling acoustic stimulus. The output from the load-cell was amplified by a factor of 1000 and recorded in Adobe Audition (Adobe Systems Incorporated, San Jose, CA) *via* a Tascam US-122 external sound card (44.1 kHz sampling rate, 16-bit resolution; TEAC Professional Division, USA). Synchronisation pulses were recorded simultaneously with the signal from the load-cell; pulses of different size denoted either a ‘gap’ or ‘no gap’ presentation. The signal was low-pass filtered at 200 Hz *post hoc*.

### 2.3 Preyer reflex

**Fig. 2:**
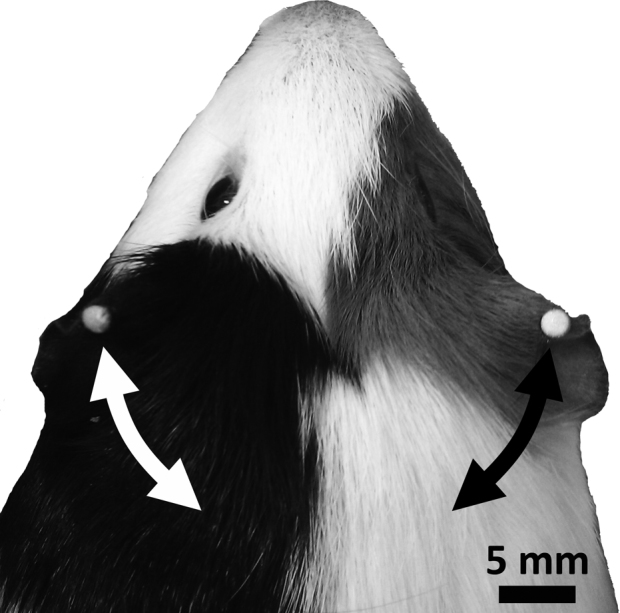
A photograph showing the position of the reflective markers when fixed to the pinnae. Arrows indicate the direction of movement of the pinna in response to a startling auditory stimulus.

In addition to measuring the WBS response, we used a motion-tracking camera system to monitor flexion of the pinna. The motion tracking system (Vicon Motion Systems, Oxford, UK) consisted of four infrared cameras. A reflective marker (4 mm diameter) was attached to each pinna using cyanoacrylate adhesive (Fig. 2), and an additional marker was attached to a central point, usually in the middle of the back, to determine the orientation of the animal. The motion-tracking system used these markers to triangulate the position of the ears, and subsequently to track pinna movement during the presentation of startling stimuli. In order to track movement effectively, the system requires a minimum of two cameras detecting marker positions. The Vicon system was calibrated at 2–3 week intervals to ensure that the motion-tracking cameras were able to correctly establish the positions of the markers; this involved using a static marker to determine the lowest vertical level of the platform and a dynamic marker to define the range of movement. Triangulated marker positions were recorded at a sampling rate of 200 Hz using Vicon Workspace software and after each trial raw data (*x*, *y*, and *z* coordinates for each marker over the time-course of a trial) were exported to Matlab^©^ (R2009b, MathWorks, MA, USA) for analysis.

### 2.4 Data analysis

Raw data, encompassing *x*, *y* and *z* coordinates for each of the markers, were exported from the Vicon motion-tracking software into an Excel compatible file (.csv). Preyer reflex data were then analysed using custom-written Matlab^©^ software. From these, the absolute positions and Euclidean distance between the markers were calculated. The centre point marker was not used for analysis; this marker was required solely for the purpose of identifying the orientation of left and right pinna markers. Marker positions were then matched to synchronisation pulses included in the sound files. The software was programmed to plot each individual startle response; this enabled manual adjustments for occasional lag between sound presentation and recording of Preyer responses (up to 1 s). In some trials, marker tracking was disrupted by GP movement occluding the markers from the camera field-of-view. Consequently, the analysis software was designed to remove startle presentations in which occlusion errors occurred during the 500 ms recording epoch, to avoid distorting the data. The magnitude of the Preyer reflex was calculated for all error-free trials as pinna displacement (change in peak-to-peak distance between right and left pinnae).

The WBS reflex was quantified as the root mean squared (RMS) amplitude of the startle-evoked response divided by the RMS amplitude immediately prior to presentation of a startle to give a ‘*amplitud*e *ratio*’ value (epochs for calculating the RMS values were 150 ms). This accounted for spontaneous movement on the platform. The RMS of the startle evoked response was calculated from 50 ms to 200 ms after the startling stimulus, *i.e.*, the time during which the WBS occurred, in order to avoid any background noise diluting the signal.

Outliers for both Preyer and WBS reflexes greater than two standard deviations from the mean were removed. For each background frequency, the mean displacement (Preyer reflex) and mean amplitude ratio (WBS) for ‘gap’ and ‘no-gap’ startle data (10 startle presentations for each condition, per trial) was calculated. A percentage difference between ‘gap’ and ‘no-gap’ data was then calculated and PPI was expressed as a percentage decrease in response when a gap was presented, compared with the ‘no-gap’ condition. Data from all sessions were pooled and the statistical significance of PPI was determined using a Wilcoxon rank-sum test to a 95% confidence rating for each GP at each background frequency. Finally, data from all GPs were pooled according to background noise condition and statistical significance compared for each reflex, with a two-way ANOVA and Bonferroni *post hoc* analysis. The variability of PPI for each reflex was assessed with a coefficient of variance test.

### 2.5 Auditory stimuli

Auditory stimuli were generated from standard 16-bit digital waveform files (.wav) files using Adobe Audition and presented through a single 25 mm loudspeaker (Peerless DX25, Tymphany, Hong Kong), *via* the Vicon motion tracking software to enable synchronisation of the onset of recording with presentation of auditory stimuli. Sound pressure level calibration was performed using a ½ inch free-field microphone (Bruel & Kjaer Model 4165) calibrated with a Bruel & Kjaer Type 42 Sound Level Calibrator. The speaker was positioned at 18.5 cm above the startle platform and aligned with the front of the cage, on its midline. The position of the GP relative to the speaker did change between, and often within, trials because the animals were not restrained. Consequently, sound levels at the animal were not always constant.

### 2.6 Measuring gap detection using PPI

The gap detection method of Turner et al. (2006) requires a continuous background noise; in this study, the background noise comprised either narrow-band noise (1 or 2 kHz bandwidth) centred at 5, 9, 13 or 17 kHz, or broadband noise. Startling stimuli were broadband noise bursts (20 ms; rise/fall time of 1 ms). A single trial consisted of 10 presentations of the startle stimulus preceded by a gap, and 10 presentations without a gap (randomised order of presentation), delivered sequentially for a given background noise condition. The inter stimulus interval (ISI) was optimised by pilot experiments to 15 or 24 s (see Section 3), leading to a single trial taking around 6 min and 30 s. The background noise was not switched off (apart from during prepulse gap presentation) during a trial. A gap duration of 50 ms (rise/fall time of 2 ms) and a delay of 100 ms between the gap onset and the startle stimulus onset were selected for all GPs included in the present study. Previous work shows that the size of the WBS response is susceptible to variations in both gap duration and delay between the gap and startling stimulus; the values selected in this study were demonstrated to be optimal for maximising gap detection ability (Friedman et al., 2004; Leitner et al., 1993). In a single testing session, each background noise condition was presented once, in a randomised order, with ∼2 min of silence between each trial. In pilot experiments, the startle stimulus was presented at either 105 or 117 dB SPL, in combination with background noise delivered at 70 dB SPL. In the main experiment the levels were chosen optimally for each GP using a sound level-dependency test (SLDT).

### 2.7 Sound level-dependency test (SLDT)

To avoid saturation of whole-body or Preyer responses, or habituation, we devised a sound level-dependency test (SLDT) protocol by which to best match the sound level of the background and startle stimuli to achieve optimal gap detection. To do this, a number of combinations of startle sound levels (95, 100, or 105 dB SPL) and narrow-band background noise (4–6 kHz) sound levels (55, 60, or 70 dB SPL) were presented, and PPI quantified. Narrow-band background noise was selected for the SLDT, as we found in earlier experiments that lower degrees of PPI were elicited when presenting narrow-band background noise, compared with BBN, and this was consistent with previous data from others (Turner and Parrish, 2008). The startle/background sound levels for optimal gap detection were selected based on the combination that produced the greatest magnitude of startle response and largest amount of PPI.

Each GP included in the main experiment (“*Comparing PPI of WBS and Preyer reflexes*”) was subjected to the SLDT, prior to commencing baseline testing. The introduction of the SLDT served a dual purpose: Firstly, determining if GPs exhibited significant PPI enabled us to discard animals that did not, before undertaking time-consuming baseline testing. Secondly, by optimising gap detection at this stage, the probability of retaining robust PPI throughout the duration of behavioural testing was increased. SLDT, as a precursor to extended measurement of PPI, is important as the magnitude of the startle response can significantly decrease after noise exposure. This is more than likely due to a reduced ability to detect the startle-eliciting stimulus (Longenecker and Galazyuk, 2011). However, it should be noted that, in the present study – owing to the improved reliability of detecting a sustained Preyer reflex (with consistently higher SNR) over WBS in our pilot experiments – the SLDT focussed on determining optimal sound levels for the Preyer reflex.

### 2.8 Comparing PPI of WBS and Preyer reflexes

Gap detection was assessed in a further group of GPs (*n* = 12) using both Preyer and WBS reflexes, and the data obtained for each GP examined for robust and consistent evidence of PPI. These GPs were first subjected to the SLDT, before undergoing testing sessions over a period of two weeks (minimum of three and a maximum of six sessions). Based on data acquired in our initial pilot experiments (see Section 3), an ISI of either 15 or 24 s (necessary to prevent short-term habituation) and a background noise bandwidth of 2 kHz, were used as optimal values for maintaining reliable startle responses and PPI.

### 2.9 Sodium salicylate

The effects of sodium salicylate on PPI of the Preyer reflex and WBS were investigated in an additional subset of GPs (*n* = 4). Mean baseline PPI was assessed in each GP (minimum three sessions/maximum six sessions over two weeks), prior to administration of sodium salicylate (350 mg kg^−1^; i.p.) dissolved in saline. Behavioural and neurophysiological effects of salicylate, when administered at this dose, have been demonstrated previously in GPs (Norena et al., 2010). PPI was subsequently measured at 2 h and 5 h post-injection, and again at 72 h to establish whether PPI had recovered to baseline levels. Data from all animals were pooled and the effects of salicylate on the Preyer and WBS responses were assessed statistically for each background noise condition at each time point with a two-way ANOVA and Bonferroni *post hoc* test. It has previously been demonstrated that the significant decrease in amplitude of the WBS following noise exposure, owing to reductions in hearing thresholds, may render changes in PPI difficult to interpret (Lobarinas et al., 2012). To account for this in our interpretation, raw amplitudes for both reflexes were analysed before and 2 h following sodium salicylate injection.

## 3 Results

The results reported here were obtained from a number of pilot studies designed to determine the optimal parameters for applying the gap detection method to the Preyer reflex, followed by the main experiment testing the efficacy of the measurement of PPI in guinea pigs.

### 3.1 Habituation of startle responses

Pilot experiments were conducted to examine the effects of randomly varying the inter-stimulus interval (ISI) between startle stimuli on the magnitude of startle responses, and habituation of PPI. In these pilot experiments two ISI values were used at random in a trial. The values used were 9 or 15 s, 12 or 20 s, and 15 or 24 s (*n* = 3 GPs). In the same animals, the effects of varying the bandwidth of the narrow-band background noise (either 1 or 2 kHz) were also assessed. Startle responses were most robust (and habituation was consequently least) with an ISI of 15 or 24 s, and a noise bandwidth of 2 kHz. It is perhaps not surprising that the longest ISI yielded the least habituation. Moreover, it is plausible that an even longer ISI would result in a further reduction of habituation. However, the duration of a testing session is a limiting factor, so ISI values of either 15 or 24 s were selected as the optimal condition.

The potential confound of either reflex habituating to startle stimuli, according to the number of trials per week, was further explored in two groups of GPs, over a five-week period: Group One was tested 2–3 times per week (*n* = 3), while Group Two was tested once a week (*n* = 5). For both groups, an ISI of either 15 or 24 s was used. No significant changes in the average magnitude of WBS and Preyer reflex startle responses were evident in either group of GPs, indicating that – when tested up to three times per week – animals did not habituate to the startle stimulus.

### 3.2 Signal-to-noise ratios and variability between reflexes

Signal-to-noise ratio (SNR) was compared for each reflex in the long-term (5-week) habituation groups of GPs, described in the previous section (*n* = 8); the mean peak-to-peak signal before (noise) and after (signal) startle presentation (500 ms epoch for each) was calculated for all ‘no-gap’ startle presentations across all trials. The SNR was calculated as 20 log (signal/noise) and expressed as dB. This process was repeated for each background noise condition, and the difference between Preyer and WBS calculated for all frequencies across all animals. The mean SNR (±SEM) of the Preyer reflex (29.2 ± 6.2 dB) was substantially higher than the SNR for WBS (21.4 ± 3.8 dB), which equated to a ∼8 dB improvement in SNR when measuring the Preyer reflex. Further to this, the variability of the startle amplitude for both reflexes was assessed with a coefficient of variance test; Preyer reflex responses exhibited ∼50% less variability than WBS.

### 3.3 Dependence of the WBS and PPI on sound level

Previous work has demonstrated a dependence of the WBS response magnitude and PPI on background and startle sound levels (Schmajuk et al., 2006). The effects of changing the sound levels of the background noise and startle stimuli were therefore assessed in a further group of animals (*n* = 5). Four GPs from the SLDT pilot group exhibited clear PPI for at least one combination of background and startle sound levels.

### 3.4 Comparing PPI of WBS and Preyer reflexes

**Fig. 3:**
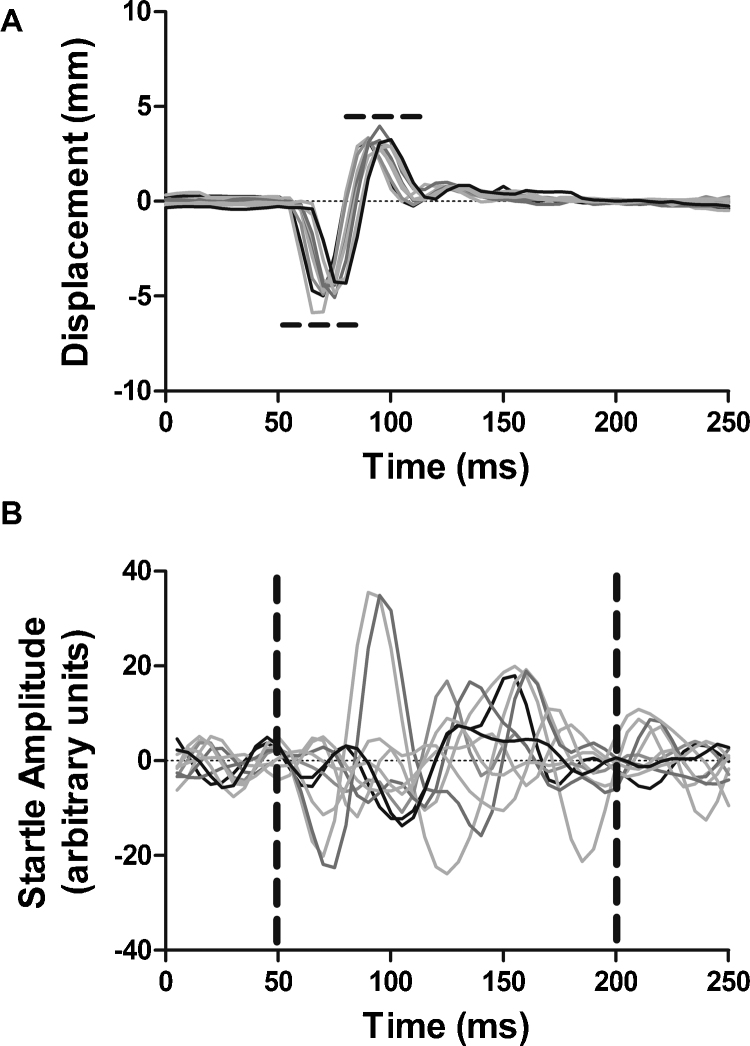
Representative raw traces of ‘no gap’ startle stimulus presentation (*n* = 10) overlaid for the Preyer reflex (A) and the WBS (B), taken from a single trial for one guinea pig. Dotted lines indicate the window of analysis for WBS and peak-to-peak measurement for Preyer reflex.

Gap detection and PPI were assessed in a group of GPs (*n* = 12) by measuring the WBS and Preyer reflexes simultaneously. Representative examples of raw recordings of the responses to a startling sound are illustrated in Fig. 3A and B. These plots are typical of the recordings we obtained for each reflex, and highlight the superior SNR and consistency (*i.e.*, minimal variability) of pinna displacement measurements (Fig. 3A) *versus* those acquired from measurements of WBS (Fig. 3B). Raw signals for all ‘gap’ stimulus presentations within a trial were pooled and mean RMS and pinna displacement plots derived for WBS and Preyer reflexes respectively; the same process was applied to ‘no gap’ presentations. PPI was then quantified for a session for each background noise condition.

PPI was assessed in each GP over 3–6 testing sessions. First, the SLDT determined whether a GP was capable of detecting a gap (and the sound levels at which this was best achieved); each animal then underwent testing sessions to obtain robust PPI. In some cases, significant PPI was obtained in three sessions (Wilcoxon rank-sum test; *P* < 0.05), but in other animals additional sessions were required (no more than six sessions – over a period of two weeks – were conducted to avoid the possible confound of habituation).

All 12 GPs exhibited significant PPI of the Preyer reflex at all background noise frequencies (*P* < 0.05). By contrast, significant PPI of the WBS reflex was only apparent at all frequencies in four GPs (see Table 1). In some cases, this was due to a complete absence of PPI at a given frequency, and in others to a much higher degree of variability in the WBS response. This was confirmed by a coefficient of variance test; the mean variation (for ‘no gap’ presentations across all frequencies/animals) of the WBS reflex was 63%, whereas the coefficient of the Preyer reflex was 39%.

**Fig. 4:**
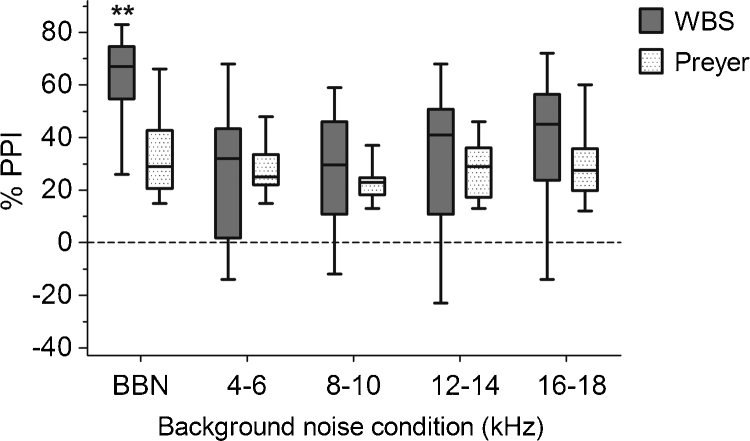
PPI of the WBS and Preyer reflexes. Mean PPI of each reflex at a given background noise frequency is indicated by the solid horizontal line contained within each box; boxes indicate 95% confidence intervals; whiskers indicate the full range of values obtained across all GPs (*n* = 12) for each condition. PPI of WBS was significantly higher than the Preyer reflex in the BBN condition (**P < 0.01).

When PPI data were pooled across animals and statistically assessed with a two-way ANOVA, significant differences were detected in both background noise centre frequency (*F*_(4,4)_ = 4.48, *P* < 0.01) and reflex type (*F*_(1,4)_ = 11.86, *P* < 0.01) variables, and a significant frequency by reflex interaction was also observed (*F*_(4,55)_ = 4.00, *P* < 0.01). *Post hoc* analysis indicated significantly higher PPI of the WBS reflex in the BBN condition, compared with the Preyer reflex (*t* = 4.92, *P* < 0.01), but no significant differences were apparent at other frequencies (Fig. 4). However, given the limited number of animals demonstrating significant PPI of WBS at all frequencies and the greater range in magnitude of PPI, the WBS measure did not appear to be optimal for detecting tinnitus perceived as tonal rather than as a broad band noise.

### 3.5 The effects of sodium salicylate on PPI

**Fig. 5:**
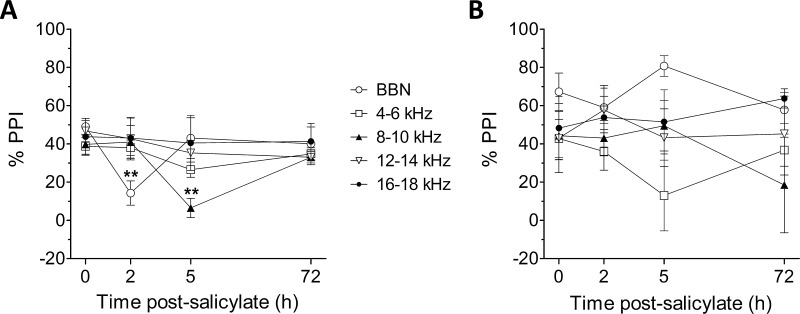
The effects of sodium salicylate on PPI of the Preyer reflex (A) and the WBS (B). Mean (± SEM) PPI values for all GPs (*n* = 4) are shown for each background frequency noise condition at 2 h, 5 h, and 72 h post-salicylate administration. For the Preyer reflex, significant reductions in PPI were seen at 2 h in the BBN condition and at 5 h in the 8–10 kHz condition (**P < 0.01). For the WBS, no significant changes were observed at any time point.

In order to compare the efficacy of detecting tinnitus using the Preyer and WBS responses, as measured by reductions in PPI, we administered sodium salicylate to four GPs and measured PPI of both reflexes at 2 h and 5 h post-injection (Fig. 5). Using the Preyer reflex as a measure of PPI (Fig. 5A), statistical analysis revealed a significant effect in the time-point variable (*F*_(3,12)_ = 3.88, *P* < 0.05), no effect in the background noise centre frequency variable (*F*_(4,12)_ = 0.73, *P* = 0.59), but a significant time by frequency interaction (*F*_(12,45)_ = 2.76, *P* < 0.01). *Post hoc* analysis indicated that salicylate significantly attenuated PPI of the Preyer reflex in the BBN background noise condition at 2 h (*t* = 3.98, *P* < 0.01) and in the 8–10 kHz background noise condition at 5 h (*t* = 3.81, *P* < 0.01). Conversely, no significant reductions in PPI of the WBS (Fig. 5B) were observed following salicylate administration for either the time-point (*F*_(3,12)_ = 0.28, *P* = 0.84) or background noise centre frequency (*F*_(4,12)_ = 1.56, *P* = 0.24) variables, or a time-frequency interaction (*F*_(12,45)_ = 1.33, *P* = 0.24).

PPI was also measured at 72 h post-salicylate administration to establish whether the effects of salicylate were transient; previous work demonstrated wash-out of salicylate effects occurring within a 72 h period (Mongan et al., 1973). No significant deficits in PPI of the Preyer reflex or WBS were apparent at this time-point in any of the background noise conditions. The deficits in PPI of the Preyer reflex may be indicative of tinnitus and the transient nature is in agreement with the predicted time-course of salicylate-induced tinnitus.

### 3.6 Changes in reflex amplitudes following salicylate administration

**Fig. 6:**
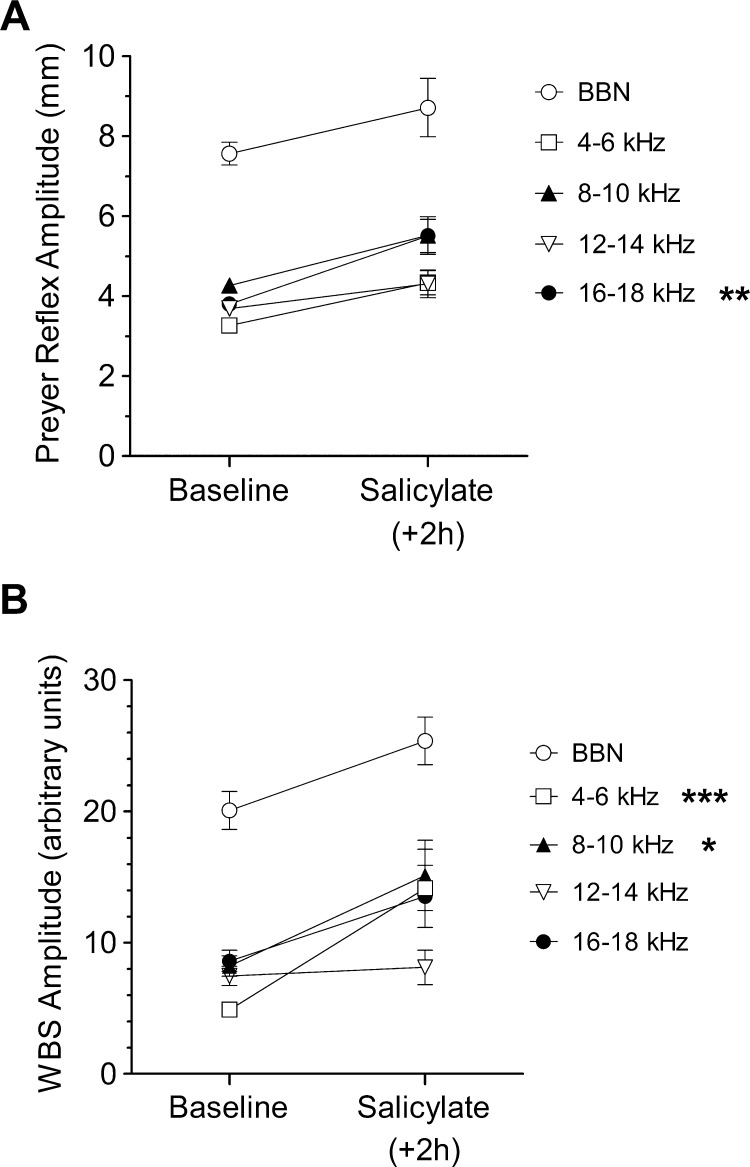
Changes in amplitude of the Preyer (A) and WBS (B) responses. Mean (± SEM) values are shown for all guinea pigs (*n* = 4) at each background noise frequency before (baseline) and 2 h after salicylate administration. Significant increases in response amplitude were detected at 16–18 kHz (**P < 0.01) for the Preyer reflex, and 4–6 and 8–10 kHz (*P < 0.05; ***P < 0.0001) for the WBS.

To determine whether the effects seen here may be purely related to changes in the amplitudes of either reflex following salicylate administration, raw Preyer reflex and WBS amplitudes were analysed before and 2 h after injection (Fig. 6). As opposed to the decreases observed following noise exposure (Lobarinas et al., 2012), both reflexes exhibited increases in startle amplitudes at all frequencies 2 h following the salicylate injection, indicating that the reductions seen in the PPI of the Preyer reflex were not simply a result of reduced startle amplitudes.

## 4 Discussion

In the present study, we have shown a novel method for quantifying PPI in GPs. This method appears to give robust consistent responses, and was more reliable than the WBS approach in our GPs. We also demonstrated sensitivity of the Preyer reflex method for detecting deficits in PPI induced by sodium salicylate, indicative of a tinnitus-like percept in our animals.

Our data indicate that the Preyer reflex was a more suitable measure than the WBS in detecting tinnitus following salicylate administration. This may be due to the variability of the WBS response in guinea pigs, as highlighted in this study. Since the development of the gap detection model by Turner et al. (2006), the WBS model has been widely used in rats (Wang et al., 2009; Yang et al., 2007), but very few groups have to date successfully induced and measured tinnitus in mice (Turner et al., 2012) or GPs (Dehmel et al., 2012a,b). GPs are notoriously difficult to train without aversive stimuli (Agterberg et al., 2010), and our early pilot studies also indicated rapid habituation to the startle stimulus, or WBS responses that were absent altogether, which further complicates adaptation of this model for use in GPs. Consequently, we were prompted to find a novel, more-robust method of evaluating PPI in the GP, while still retaining the essential characteristic – measurement of a reflex requiring no training. The Preyer reflex (described in Bohmer, 1988) appears to offer an elegant solution to these limiting factors. In the present study we have demonstrated robust, reproducible startle-evoked responses that appear far less susceptible to habituation, exhibit a superior SNR, and show clear PPI, when compared with our WBS data. Most importantly, we were able to demonstrate baseline PPI of the Preyer reflex at all background noise frequencies in all twelve GPs tested, whereas this was only the case in four of the GPs when evaluating WBS. Moreover, PPI of the Preyer reflex was sensitive to manipulations with sodium salicylate whereas the WBS was not, and thus may present a useful alternative for relating changes in a reflex response to tinnitus. Although we measured the Preyer reflex using expensive motion tracking hardware, there is little reason why the reflex could not be measured using simpler hardware such as accelerometers.

Salicylate causes transient and reversible tinnitus when administered at high doses in both humans (Mongan et al., 1973) and animals (Ralli et al., 2010). Behaviourally, salicylate has been shown to significantly impair gap detection in rats (Turner and Parrish, 2008), consistent with the results shown here. Although the precise mechanisms behind salicylate-induced tinnitus have not – as yet – been elucidated, a number of neural changes have been observed following salicylate administration in animal experiments (Evans and Borerwe, 1982; Eggermont and Kenmochi, 1998; Basta and Ernst, 2004; Lobarinas et al., 2006; Roberts et al., 2010; Wei et al., 2010). The pathways for tinnitus generation are different dependent on the inducing agent. Nonetheless, salicylate is a useful tool for examining tinnitus. Salicylate treatment reliably induces transient tinnitus; this is proposed (at the central level) to occur as a result of decreased GABAergic transmission and a subsequent loss of inhibition (see Stolzberg et al., 2012 for review).

An important, and potentially confounding, aspect of the gap detection method following tinnitus induction is a reduction in startle amplitudes that has previously been observed after noise exposure (Lobarinas et al., 2012). The potential implication of this finding is that such a decrease may render any gap detection deficits difficult to interpret, as the PPI calculation is a relative measure and thus could be affected by reduced startle amplitudes. In light of the greater degree of variability as well as the poorer SNR of the WBS (compared with Preyer) in our data, it is conceivable that any reduction in amplitudes may often obscure a detectable startle response and render calculations of PPI as meaningless.

In the present study, we did not observe any reductions in the amplitudes of either Preyer or WBS responses following salicylate administration. In fact, significant increases were present at various background frequencies. It has previously been suggested that such a finding may occur as a result of hyperacusis (Sun et al., 2009), reflecting oversensitivity to the startling stimulus. However, it is important to highlight that whilst startle amplitudes did not decrease following salicylate administration, decreased amplitudes are clearly prevalent following noise exposure (Lobarinas et al., 2012). Consequently, any future studies using the gap detection method, regardless of which startle response is measured, should ensure that a clearly detectable response is analysed following noise exposure. Furthermore, hearing thresholds should have recovered sufficiently in order for an animal to detect and respond to the background stimulus, as this may also confound interpretation of gap detection deficits.

Although the advantages of using the Preyer reflex were clear from our data (three times as many GPs demonstrating baseline PPI at every frequency and sensitivity to changes following salicylate administration), an important caveat lies in the design of the SLDT protocol used in these experiments. The SLDT was conducted to determine the optimal sound level combinations (background/startle) for detecting PPI of the Preyer reflex not the WBS. However, from our early pilot experiments that showed inferior SNR and lower levels of detectable WBS, it still seems likely that the Preyer reflex provides a ‘cleaner’, more-robust measure in our GPs. The WBS reflex has previously been shown to successfully identify tinnitus in naturally active species, such as rats (Turner et al., 2006) and mice (Turner et al., 2012), but the Preyer reflex may be more suitable in more lethargic animals such as chinchillas or cats (Koka et al., 2011). Furthermore, these animals have large pinnae which would render any Preyer response easier to detect; this may be an important factor in the utility of this approach to measuring gap detection. It would be useful to compare the two methods for measuring PPI in other species.

There are limitations to using the reflex response approach that at present remain unresolved. In either form, the method measures – at best – perception of a phantom sound, but fails to assess the emotional components and characteristics that are linked to the level of annoyance produced in the human condition. This, clearly, is a challenging facet to model and quantify in animals, while retaining other features of the current models and not introducing complex behavioural tasks that may interfere with measurement of the phantom sound perception. Despite recent advances in animal tinnitus models, the scope for understanding the pathophysiology of tinnitus remains limited without significant further development. We are currently trying to find electrophysiological and histological markers of tinnitus that would provide complementary evidence for making a more secure diagnosis.

## Figures and Tables

**Table 1:**
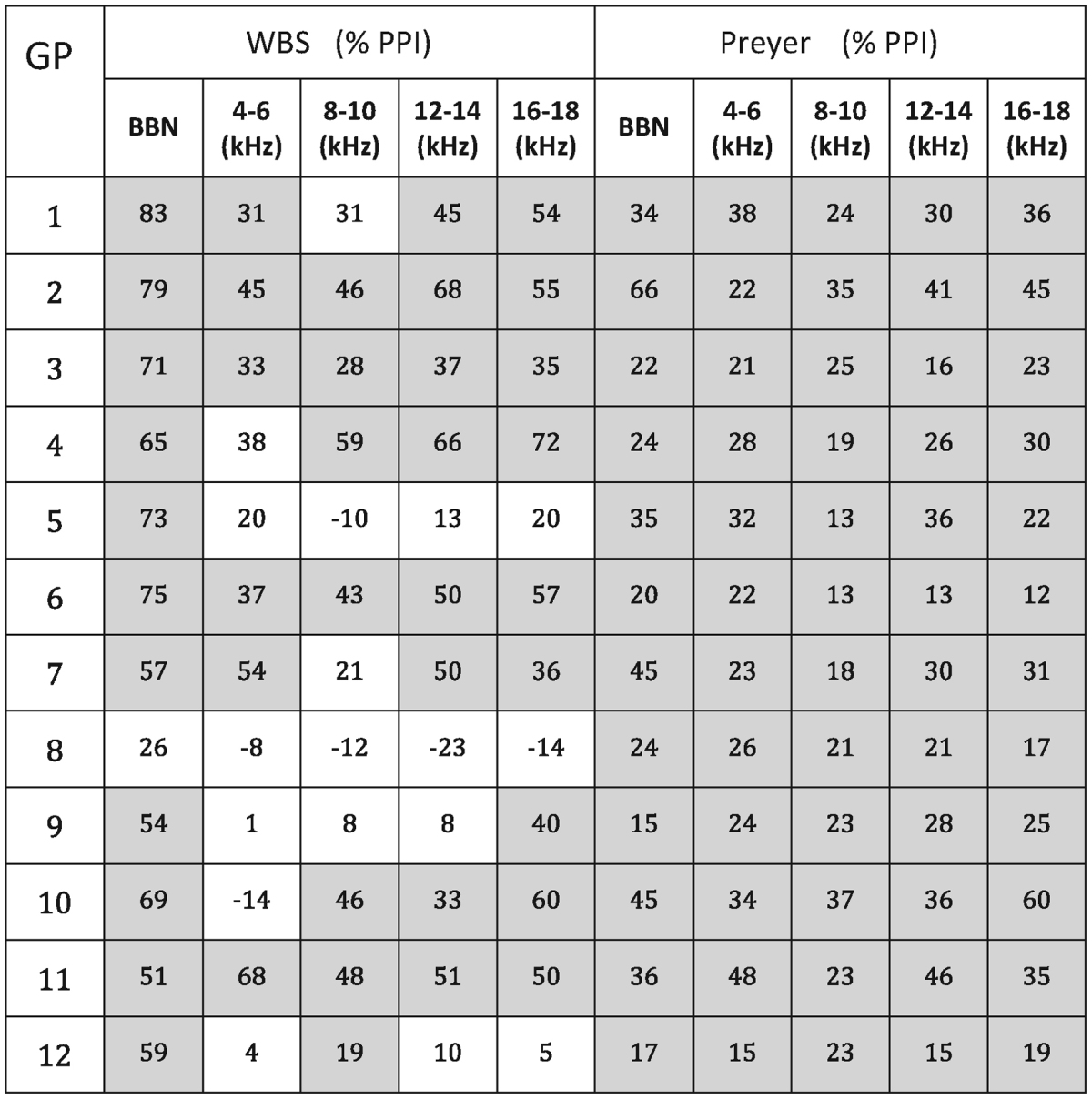
PPI of the WBS and Preyer reflexes at each background noise frequency (BBN, 4–6 kHz, 8–10 kHz, 12–14 kHz, 16–18 kHz), expressed as % PPI, are shown for all GPs (*n* = 12). Shading and emboldened text indicates significant PPI values, as determined with a Wilcoxon rank-sum test (*P* < 0.05). All GPs demonstrated significant PPI of the Preyer reflex, whereas only four GPs (GP2, GP3, GP6, and GP11) showed significant PPI of the WBS reflex at all frequencies.

## References

[bib0005] Agterberg M.J., van den Broek M., Philippens I.H. (2010). A less stressful animal model: a conditioned avoidance behaviour task for guineapigs. Lab Anim.

[bib0010] Basta D., Ernst A. (2004). Effects of salicylate on spontaneous activity in inferior colliculus brain slices. Neurosci Res.

[bib0015] Bauer C.A., Brozoski T.J. (2001). Assessing tinnitus and prospective tinnitus therapeutics using a psychophysical animal model. J Assoc Res Otolaryngol.

[bib0020] Bohmer A. (1988). The Preyer reflex – an easy estimate of hearing function in guinea pigs. Acta Otolaryngol.

[bib0025] Cassella J.V., Davis M. (1986). Habituation, prepulse inhibition, fear conditioning, and drug modulation of the acoustically elicited pinna reflex in rats. Behav Neurosci.

[bib0030] Cazals Y. (2000). Auditory sensori-neural alterations induced by salicylate. Prog Neurobiol.

[bib0035] Davis M., Falls W.A., Campeau S., Kim M. (1993). Fear-potentiated startle: a neural and pharmacological analysis. Behav Brain Res.

[bib0040] Dehmel S., Eisinger D., Shore S.E. (2012). Gap prepulse inhibition and auditory brainstem-evoked potentials as objective measures for tinnitus in guinea pigs. Front Syst Neurosci.

[bib0045] Dehmel S., Pradhan S., Koehler S., Bledsoe S., Shore S. (2012). Noise overexposure alters long-term somatosensory-auditory processing in the dorsal cochlear nucleus – possible basis for tinnitus-related hyperactivity?. J Neurosci.

[bib0050] Eggermont J.J., Kenmochi M. (1998). Salicylate and quinine selectively increase spontaneous firing rates in secondary auditory cortex. Hear Res.

[bib0055] Eggermont J.J., Roberts L.E. (2004). The neuroscience of tinnitus. Trends Neurosci.

[bib0060] Evans E.F., Borerwe T.A. (1982). Ototoxic effects of salicylates on the responses of single cochlear nerve fibres and on cochlear potentials. Br J Audiol.

[bib0065] Friedman J.T., Peiffer A.M., Clark M.G., Benasich A.A., Fitch R.H. (2004). Age and experience-related improvements in gap detection in the rat. Brain Res Dev Brain Res.

[bib0070] Harrison R.V., Aran J.M., Erre J.P. (1981). AP tuning curves from normal and pathological human and guinea pig cochleas. J Acoust Soc Am.

[bib0075] Heffner H.E., Harrington I.A. (2002). Tinnitus in hamsters following exposure to intense sound. Hear Res.

[bib0080] Heller A.J. (2003). Classification and epidemiology of tinnitus. Otolaryngol Clin North Am.

[bib0085] Hoffman H.S., Searle J.L. (1965). Acoustic variables in the modification of startle reaction in the rat. J Comp Physiol Psychol.

[bib0090] Jastreboff P.J., Brennan J.F., Coleman J.K., Sasaki C.T. (1988). Phantom auditory sensation in rats: an animal model for tinnitus. Behav Neurosci.

[bib0095] Jero J., Coling D.E., Lalwani A.K. (2001). The use of Preyer's reflex in evaluation of hearing in mice. Acta Otolaryngol.

[bib0100] Kaltenbach J.A. (2011). Tinnitus: models and mechanisms. Hear Res.

[bib0105] Koka K., Jones H.G., Thornton J.L., Lupo J.E., Tollin D.J. (2011). Sound pressure transformations by the head and pinnae of the adult Chinchilla (*Chinchilla lanigera*). Hear Res.

[bib0110] Leitner D.S., Hammond G.R., Springer C.P., Ingham K.M., Mekilo A.M., Bodison P.R. (1993). Parameters affecting gap detection in the rat. Atten Percept Psychophys.

[bib0115] Li L., Frost B.J. (1996). Azimuthal sensitivity of rat pinna reflex: EMG recordings from cervicoauricular muscles. Hear Res.

[bib0120] Lobarinas E., Hayes S.H., Allman B.L. (2012). The gap-startle paradigm for tinnitus screening in animal models: limitations and optimization. Hear Res.

[bib0125] Lobarinas E., Yang G., Sun W., Ding D., Mirza N., Dalby-Brown W. (2006). Salicylate- and quinine-induced tinnitus and effects of memantine. Acta Otolaryngol Suppl.

[bib0130] Longenecker R.J., Galazyuk A.V. (2011). Development of tinnitus in CBA/CaJ mice following sound exposure. J Assoc Res Otolaryngol.

[bib0140] Mongan E., Kelly P., Nies K., Porter W.W., Paulus H.E. (1973). Tinnitus as an indication of therapeutic serum salicylate levels. J Am Med Assoc.

[bib0145] Norena A.J., Moffat G., Blanc J.L., Pezard L., Cazals Y. (2010). Neural changes in the auditory cortex of awake guinea pigs after two tinnitus inducers: salicylate and acoustic trauma. Neuroscience.

[bib0150] Prosen C.A., Petersen M.R., Moody D.B., Stebbins W.C. (1978). Auditory thresholds and kanamycin-induced hearing loss in the guinea pig assessed by a positive reinforcement procedure. J Acoust Soc Am.

[bib0155] Ralli M., Lobarinas E., Fetoni A.R., Stolzberg D., Paludetti G., Salvi R. (2010). Comparison of salicylate- and quinine-induced tinnitus in rats: development, time course, and evaluation of audiologic correlates. Otol Neurotol.

[bib0160] Roberts L.E., Eggermont J.J., Caspary D.M., Shore S.E., Melcher J.R., Kaltenbach J.A. (2010). Ringing ears: the neuroscience of tinnitus. J Neurosci.

[bib0165] Schmajuk N.A., Larrauri J.A., Hagenbuch N., Levin E.D., Feldon J., Yee B.K. (2006). Startle and prepulse inhibition as a function of background noise: a computational and experimental analysis. Behav Brain Res.

[bib0170] Stolzberg D., Salvi R.J., Allman B.L. (2012). Salicylate toxicity model of tinnitus. Front Syst Neurosci.

[bib0175] Sun W., Lu J., Stolzberg D., Gray L., Deng A., Lobarinas E. (2009). Salicylate increases the gain of the central auditory system. Neuroscience.

[bib0180] Swerdlow N.R., Geyer M.A., Braff D.L. (2001). Neural circuit regulation of prepulse inhibition of startle in the rat: current knowledge and future challenges. Psychopharmacology (Berl).

[bib0185] Turner J., Larsen D., Hughes L., Moechars D., Shore S. (2012). Time course of tinnitus development following noise exposure in mice. J Neurosci Res.

[bib0190] Turner J.G., Brozoski T.J., Bauer C.A., Parrish J.L., Myers K., Hughes L.F. (2006). Gap detection deficits in rats with tinnitus: a potential novel screening tool. Behav Neurosci.

[bib0195] Turner J.G., Parrish J. (2008). Gap detection methods for assessing salicylate-induced tinnitus and hyperacusis in rats. Am J Audiol.

[bib0200] Wang H., Brozoski T.J., Turner J.G., Ling L., Parrish J.L., Hughes L.F. (2009). Plasticity at glycinergic synapses in dorsal cochlear nucleus of rats with behavioral evidence of tinnitus. Neuroscience.

[bib0205] Wei L., Ding D., Sun W., Xu-Friedman M.A., Salvi R. (2010). Effects of sodium salicylate on spontaneous and evoked spike rate in the dorsal cochlear nucleus. Hear Res.

[bib0210] West C.D. (1985). The relationship of the spiral turns of the cochlea and the length of the basilar membrane to the range of audible frequencies in ground dwelling mammals. J Acoust Soc Am.

[bib0215] Yang G., Lobarinas E., Zhang L., Turner J., Stolzberg D., Salvi R. (2007). Salicylate induced tinnitus: behavioral measures and neural activity in auditory cortex of awake rats. Hear Res.

